# Smoking, pregnancy and the subgingival microbiome

**DOI:** 10.1038/srep30388

**Published:** 2016-07-27

**Authors:** Akshay D. Paropkari, Binnaz Leblebicioglu, Lisa M. Christian, Purnima S. Kumar

**Affiliations:** 1 Division of Periodontology, College of Dentistry, The Ohio State University, Columbus, Ohio, USA; 2 Institute for Behavioral Medicine Research, The Ohio State University, Columbus, Ohio, USA

## Abstract

The periodontal microbiome is known to be altered during pregnancy as well as by smoking. However, despite the fact that 2.1 million women in the United States smoke during their pregnancy, the potentially synergistic effects of smoking and pregnancy on the subgingival microbiome have never been studied. Subgingival plaque was collected from 44 systemically and periodontally healthy non-pregnant nonsmokers (control), non-pregnant smokers, pregnant nonsmokers and pregnant smokers and sequenced using 16S-pyrotag sequencing. 331601 classifiable sequences were compared against HOMD. Community ordination methods and co-occurrence networks were used along with non-parametric tests to identify differences between groups. Linear Discriminant Analysis revealed significant clustering based on pregnancy and smoking status. Alpha diversity was similar between groups, however, pregnant women (smokers and nonsmokers) demonstrated higher levels of gram-positive and gram-negative facultatives, and lower levels of gram-negative anaerobes when compared to smokers. Each environmental perturbation induced distinctive co-occurrence patterns between species, with unique network anchors in each group. Our study thus suggests that the impact of each environmental perturbation on the periodontal microbiome is unique, and that when they are superimposed, the sum is greater than its parts. The persistence of these effects following cessation of the environmental disruption warrants further investigation.

The oral cavity plays host to a large and diverse group of bacteria; which form biofilm communities in several habitats within the mouth, including the tooth, subgingival sulcus, tongue, buccal mucosa and tonsils^1^. Thus, the oral cavity may be regarded as a collection of geographically distinct yet interconnected microbial ecosystems. Host-associated microbial communities play important roles in maintaining health. Several mechanisms have emerged in the recent literature, such as niche saturation, colonization resistance, prevention of pathogen expansion, nutritional and structural symbiosis, host immune education and metabolic support^2^. It has been established that, especially in the oral cavity, loss or reduction of health-compatible species creates dysbiosis within specific ecosystems^3^, thereby leading to periodontal disease, caries and oral cancer^4^. The composition of a microbial community depends on several factors, some of which are related to host genotype (for example, gender, ethnicity, dentition, tooth morphology) and environmental factors (for example, diet, smoking and oral hygiene habits)^5^,^6^,^7^,^8^,^9^,^10^. While the composition of health-associated periodontal communities has been well studied, little is known about the impact of environmental factors in shaping these indigenous biofilms in states of health.

Bacteria form biofilms in the subgingival habitat soon after the tooth erupts; and a dynamic equilibrium between the subgingival microbiome and the host immune system is a critical determinant of periodontal health (reviewed by Kumar *et al.*^2^). In any ecosystem, two types of environmental stimuli can impact bacterial colonization and growth. A pressed event is defined as one which, when initiated, stays in place for a long time; while a pulsed perturbation is one that has a sudden onset, is of short duration in comparison to the time span under consideration and may be repeated^10^.

An example of a pulsed perturbation that affects only females is pregnancy. The association between pregnancy and subgingival bacteria has been examined using cultivation, microscopy, DNA-DNA checkerboard and quantitative real-time PCR^11^,^12^,^13^,^14^,^15^. While earlier studies implicated certain species collectively known as black-pigmented Bacteroides (BPB) in the etiopathogenesis of pregnancy gingivitis, open-ended studies have been equivocal regarding the effect of pregnancy on the abundance of these species. Although *in vitro* studies have demonstrated that BPB use estrogen as naphthoquinone substitutes for respiration, it is not clear from the human studies if the levels of BPB is higher in pregnant females due to the inflammatory state or due to hormonal influence^16^. More importantly, it has been estimated that 10% of women (1.2 million) smoke during their pregnancy (www.cdc.gov/prams/pramstat.htm).

Earlier studies from our laboratory and others have highlighted the role of an environmental press - cigarette smoking - in changing the oral microbiome; by decreasing the levels of beneficial species, and promoting a pathogen-rich microbial community within 24-hours of biofilm formation^8^,^12^,^17^,^18^,^19^ thereby increasing the risk for periodontitis.

Since there is a robust body of evidence to support the individual impacts of these two perturbations on the subgingival microbiome, the purpose of the present investigation was to examine the combined effects smoking and pregnancy in shaping the subgingival microbiome using high-resolution, high-throughput approaches.

## Materials and Methods

### Study population and sample collection

Approval for this study was obtained from the Office of Responsible Research Practices at The Ohio State University and the study was conducted in accordance with the approved guidelines. Women who were 18–35 years of age and between 21–24 weeks of gestation were recruited during their regular visits to The Ohio State University Wexner Medical Center Prenatal Clinic between March 2010 and May 2011 and informed consent obtained. Age-matched non-pregnant women were recruited from the Dental Clinics of The Ohio State University and informed consent obtained. Subjects had to have at least 20 teeth, periodontal health (CAL ≤1 mm, less than 3 sites with 4 mm of probe depths (PD), bleeding index (BOP) ≤30%), no antibiotics or professional prophylaxis for at least 3 months. Exclusion criteria were carrying more than one fetus during current pregnancy and previous history of miscarriage and/or preterm delivery. Also, women who had health conditions that affect immune or endocrine functions, including diabetes, hypertension, thyroid disorders and women with heart conditions that would require antibiotic prophylaxis prior to dental visits were excluded. Women with asthma and arthritis who required regular use of anti-inflammatory medications were also excluded. In addition, subjects with a history of alcohol/drug abuse and women who were using mood-altering medications were excluded. Inclusion criteria for smokers were 5 pack years or greater of tobacco exposure, and nonsmokers were defined as individuals who had smoked less than 100 cigarettes in their lifetime and were currently not smoking (CDC guidelines). Clinical examination was conducted by a single calibrated periodontist. Prior to clinical examination, maxillary anterior teeth from right second premolar to left second premolar were isolated and dried. Sterile periodontal paper strips (OraFlow, Hewlett, NY) were gently inserted into buccal interproximal areas of teeth without BOP for 30 seconds. Strips were pooled for each subject and stored at −80 °C until further analysis.

### DNA isolation and sequencing

Paper strips were separated from waxed portion using sterile scissors, 200 μl of sterile cold phosphate buffered saline added and centrifuged. Bacterial DNA was isolated from 100 μl of eluent with a Qiagen DNA MiniAmp kit (Qiagen, Valencia, CA, USA) using the tissue protocol according to the manufacturer’s instructions.

### Sequencing and data analysis

Multiplexed bacterial tag-encoded FLX amplicon pyrosequencing was performed using the Titanium platform (Roche Applied Science, Indianapolis, IN, USA) as previously described^20^ in a commercial facility (MRDNALab. Shallowater, TX, USA). Briefly, a single step PCR with broad-range universal primers and 22 cycles of amplification was used to amplify the 16S rRNA genes as well as to introduce adaptor sequences and sample-specific bar-code oligonucleotide tags into the DNA. Two regions of the 16S rRNA genes were sequenced: V1–V3 and V7–V9. The primers used for sequencing have been previously described^21^. Adaptor sequences were trimmed from raw data with 98% or more of bases demonstrating a quality control of 30 and sequences binned into individual sample collections based on bar-code sequence tags, which were then trimmed. Sequences <300 bp were discarded and the rest were clustered into species- level operational taxonomic units (s-OTUs) at 97% sequence similarity and assigned a taxonomic identity by alignment to locally hosted version of the HOMD database using the Blastn algorithm. Analyses were conducted using the QIIME pipeline^22^, as well as our own internally developed analysis pipeline PhyloToAST^23^. Results were visualized using the Python library *matplotlib*. Phylogenetic tree data was visualized through the Interactive Tree Of Life webserver^24^.

### Statistical analysis

LDA was implemented with *MASS* package in R. MASS:lda provided singular value decomposition (SVD) values, which were used to calculate variances in each dimension. Packages *scatterplot3d* and *rgl* were used to visualize 3D LDA plots. The input for LDA was a matrix of normalized (arc-sine transformed) relative abundances of species-level OTUs. Single and multiple comparisons of distributions were carried out with the statistical facilities provided by JMP (SAS Institute Inc.), as well as the Python libraries *SciPy*, *pandas*, and *statsmodels*. Statistical significances of s-OTUs within the four groups were determined using paired Wilcoxon’s tests. Significant pairwise correlations (p < 0.05, Spearman’s ρ) and graph theory were used to compute species-level co-occurrence networks for each group. Network graphs were calculated using *Networkx* package in Python and visualized in *Gephi*^25^.

### Identifying network anchors

Network anchors were identified using an algorithm incorporating betweenness centrality, differential abundances, and frequency of occurrence in a group. Betweenness centrality was calculated using Python package “*Networkx”* and s-OTUs were ranked based on this metric. For each group, significantly different (p < 0.05, Tukey-HSD) species were identified using JMP (SAS Institute Inc.) and species that were present in at least 75% of the subjects were identified using QIIME’s core_microbiome.py script. Species that demonstrated a high betweenness centrality (top 20% in each network), and were either part of the group’s core microbiome or showed significant differences for the groups were identified as network anchors.

## Results

A total of 44 periodontally and systemically healthy women between the ages of 18 and 25 were recruited (Table 1). 80% of the study population was African American. The age and race distribution among four groups were similar. In addition, the number of smokers and tobacco exposure within pregnant and non-pregnant groups was also similar.

**Figure 1:**
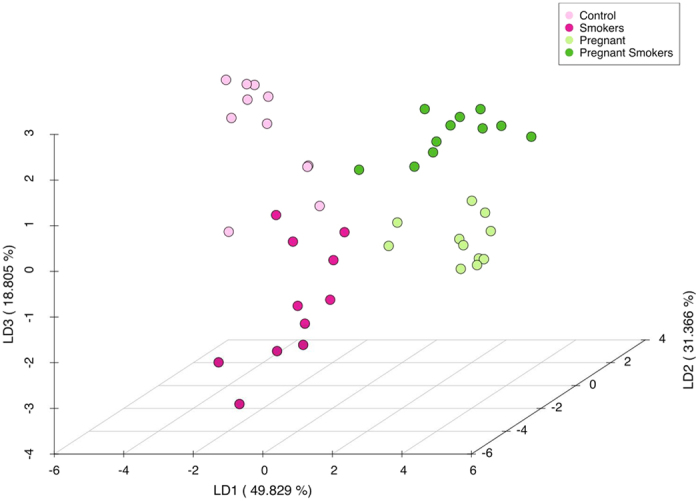
Dissimilarity in microbial community configuration between the groups. Linear discriminant analysis of relative abundances of species-level operational taxonomic units (s-OTUs) is shown. The microbial profiles of subjects clustered by pregnancy and smoking status, creating four statistically significant clusters (p < 0.05, MANOVA/Wilks).

331601 classifiable sequences were used for analysis. These sequences represented 218 species belonging to 116 genera. Each individual was colonized by 112 ± 18 species. Statistically significant group separation was evident among all groups (p < 0.001, MANOVA/Wilks lambda). The variance expressed by first, second and third linear discriminant (LD) was 49.83%, 31.37%, and 18.81%, respectively. LD1 discriminated between pregnant and non-pregnant women groups, LD2 discriminated between smokers and control groups and LD3 discriminated between smokers and nonsmokers in the pregnant group. (Fig. 1).

**Figure 2:**
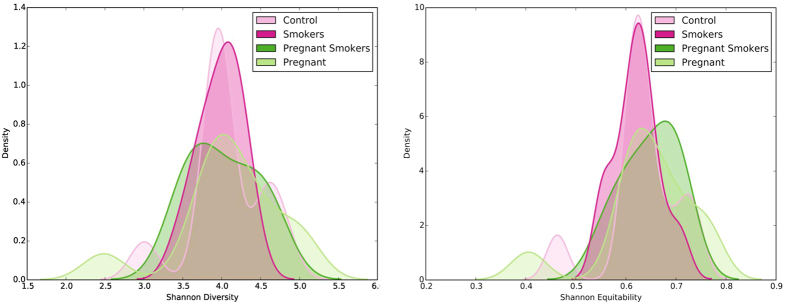
Alpha diversity and equitability in the four groups. Kernel plot of density curves for Shannon Diversity Index in non-pregnant, non-smoking controls, pregnant women, smokers and pregnant smokers are shown in 2A, while the same plots of Shannon Equitability Index are shown in Fig. 2B. The index was not significantly different between groups (p > 0.05, Wilcoxon signed rank test).

Alpha diversity ranged from 1.75 to 5.75 (Shannon Diversity Index). Both controls and pregnant women demonstrated a bimodal distribution of values, however, for all groups the median diversity was centered around 4. This was not statistically different between the four groups (p > 0.05, Wilcoxon signed rank test Fig. 2A). The median equitability of this distribution was centered on 0.6; again, the equitability index demonstrated a bimodal distribution and was not significantly different between groups (Fig. 2B).

**Figure 3:**
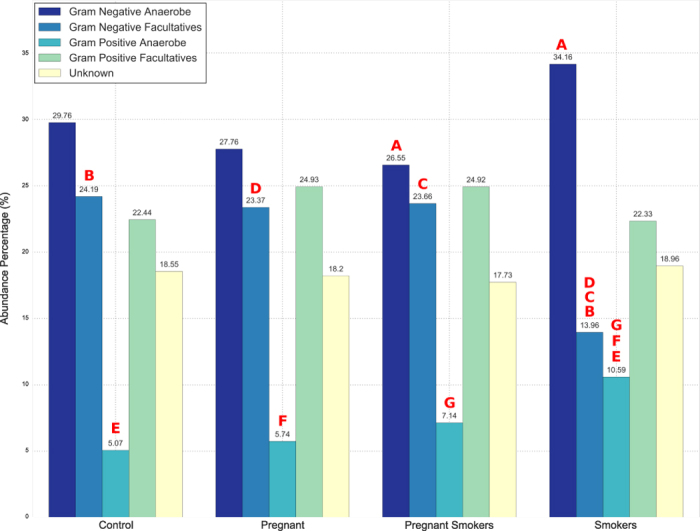
Gram staining characteristics and oxygen requirements of species. Significant differences (p < 0.05, Wilcoxon signed rank test) were observed between non-pregnant, non-smoking controls, smokers and pregnant women when the s-OTUs were stratified based on these criteria. Significance among pairwise comparison is denoted by same alphabets in red on top of bars. There were no significant differences between pregnant smokers and nonsmokers.

There was significant difference in community structure based on gram characteristics and abundances of species (Fig. 3). Probable gram staining characteristics and oxygen requirements were attributed to uncultivated species based on phylogenetic relatedness to the closest cultivated species. Smoking was associated with lower levels of gram-negative facultatives and higher levels of gram-negative anaerobes when compared to controls. Pregnancy, on the other hand, was associated with lower levels of gram-positive and gram-negative anaerobes, with higher levels of gram-negative facultatives, when compared to controls. This was seen in both smokers and nonsmokers who were pregnant. Pregnant women also demonstrated lower levels of anaerobes (gram positive and gram-negative) and higher levels of gram-negative facultatives when compared to non-pregnant smokers (p < 0.05, Wilcoxon signed rank test).

**Figure 4:**
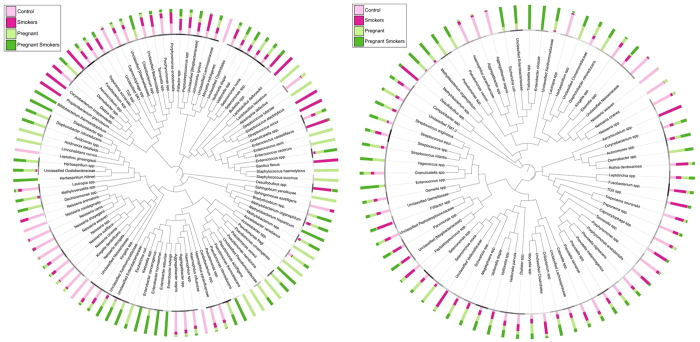
Distribution of significant species by groups. Relative abundances of s-OTUs that were significantly different between groups (p < 0.05, Wilcoxon signed rank test) are shown in Fig. 4A and those that were abundant in each group are shown in Fig. 4B.

The differences in gram staining characteristics and oxygen requirements were reflected at the species level (Fig. 4A); several s-OTUs were significantly different between groups (p < 0.05, Wilcoxon signed rank test). Species belonging to the genera *Pseudomonas, Acidovorax, Enterobacter, Enterococcus, Diaphorobacterium* and *Methylobacterium* demonstrated significantly greater abundances in pregnant women (both smokers and nonsmokers), with some of these uniquely identified in the pregnancy-associated microbiome. Both smoking and pregnancy were associated with a significant decrease in species belonging to *Neisseria* and *Aggregatibacter* when compared to controls. Smokers (non-pregnant) demonstrated significantly greater levels of species belonging to TG5, *Filifactor, Fusobacterium, Lactobacillus, Desulfobulbus, Streptococcus, Propionibacterium* and *Corynebacterium* than either pregnant women or controls. Figure 4B shows the s-OTUs that formed the core microbiome (identified in 75% or more of individuals in each group). The groups differed significantly based on their predominant microbiota. Several organisms that demonstrated significantly differing abundances between groups (Fig. 4A) were also dominant members of the core microbial community. For example, TG5, *Filifactor, Fusobacterium* and *Desulfobulbus* formed the numerical majority of the subgingival microbiome of smokers. Similarly, *Neisseria* and *Aggregatibacter* were abundant in controls, but demonstrated significantly lower levels in other groups. When pregnant smokers were compared to controls, 51 species (32% of the species found in controls or pregnant smokers) demonstrated differing abundances. Of these, 31 were also different either between smokers and controls or between pregnant women and controls.

**Figure 5:**
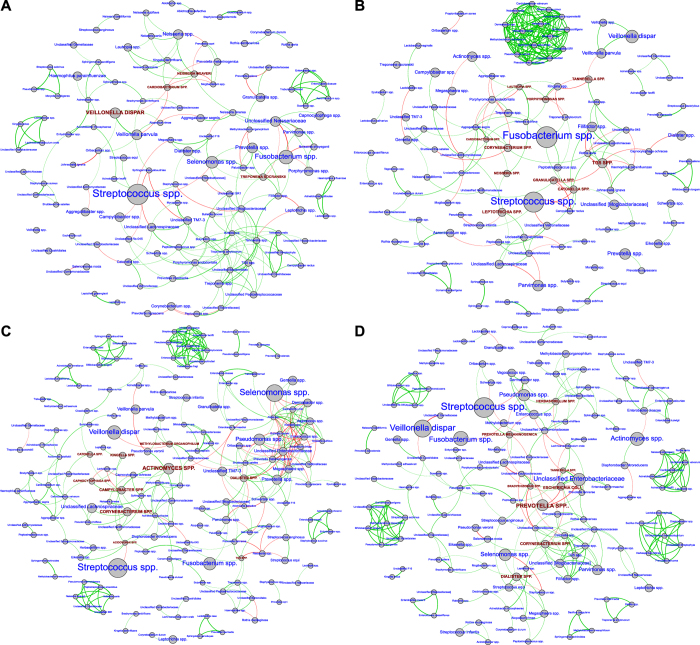
Co-occurrence networks in each group. Non-pregnant, non-smoking control group are shown in Fig. 5A, smokers in 5B, pregnant women in 5C and pregnant smokers in 5D. Each network graph contains nodes (circles sized by relative abundance per group) and edges (lines). Nodes represent species-level OTU’s and edges represent Spearman’s ρ. Edges are colored green for positive correlation and red for negative correlation. Genera contributing to network robustness (anchoring OTUs) were computed as a function of genus degree. Only significant correlations (p < 0.05, t-test) with a coefficient of at least 0.75 are shown. Network anchors are highlighted in brown font and capital lettering.

Bacteria that demonstrated significantly different abundances in each group also demonstrated differing co-occurrence patterns, Co-occurrence networks in the control group are shown in Fig. 5A, smokers in 5B, pregnant women in 5C and pregnant smokers in 5D. Each network graph contains nodes (circles) and edges (lines). Nodes represent species-level OTU’s (and are sized by relative abundance) and edges represent Spearman’s ρ. Edges are colored green for positive correlation and red for negative correlation. Only significant correlations (p < 0.05, t-test) with a coefficient of at least 0.75 are shown. Network anchors are highlighted in brown font and capital lettering. In contrast to controls, all three environmental perturbations (smoking, pregnancy and both) demonstrated multiple small tightly clustered networks. It was interesting to note that the most abundant species were not necessarily important in creating or anchoring clusters. Non-pregnant, nonsmoker control group consisted of *Cardiobacterium spp.*, *Neisseria weaveri, Treponema socranskii* and *Veillonella dispar* as significant (p < 0.05, Steel-Dwass test), part of core species group and highly ranked in betweenness centrality measure. *Cardiobacterium spp.* and *Neisseria weaveri* are also unique species of control group. In pregnant women, *Acidovorax spp.*, *Actinomyces spp., Campylobacter spp., Capnocytophaga spp., Catonella spp., Corynebacetrium spp., Kingella spp., Dialister spp.,* and *Methylobacterium organophilum* were network anchors, while in smokers *Catonella spp., Corynebacetrium spp., Cardiobacterium spp., Granulicatella spp., Lautropia spp., Leptotrichia spp., Neisseria spp., Porphyromonas spp., Tannerella spp.,* and *TG5 spp.* were important as anchors. Pregnant smokers demonstrated cluster anchors that were not seen in either pregnant women or in smokers, namely, *Bradyrhizobium spp., Herbaspirillum, E.coli, Prevotella melalinogenica,* and *Prevotella spp.,* along with *Corynebacetrium spp., Dialister spp.,* and *Tannerella spp.*

## Discussion

It has been known for a number of decades that both the prevalence and severity of gingivitis is greater in pregnant women than in non-pregnant controls^26^,^27^,^28^,^29^. Pregnant women also have greater amounts of gingival inflammation, deeper probe depths, gingival crevicular fluid levels and bleeding during all three trimesters than following parturition (reviewed by Kumar^16^). Cultivation-based investigations have demonstrated an increase in prevalence and levels of black-pigmented *Bacteroides,* particularly *Bacteroides melaninogenicus ss. intermedius,* during the second trimester of pregnancy^14^,^15^,^30^,^31^. These increases were reported to occur independent of increase in plaque levels; however, they were accompanied by an increase in gingival index and subgingival levels of anaerobic bacteria. Since it is not evident if the levels of bacteria were in response to the inflammation or the steroid surge, or both, we examined the microbiomes of individuals with minimal clinical evidence of gingival inflammation. In the present study, out of 115 pregnant women who were screened, 34 pregnant women were diagnosed with gingivitis and 69 subjects were diagnosed with chronic periodontitis, and 22 qualified based on our clinical criteria for periodontal health (delineated in the methods section).

We examined the effects of smoking and pregnancy on subgingival microbial profiles using two strategies. Principal co-ordinate analysis of UniFrac distances was initially used to examine similarities in microbial profiles between individuals. There was statistically significant clustering of individuals based on pregnancy and smoking status (Adonis test p < 0.05, data not shown), indicating that smoking and pregnancy promote colonization by genetically distinct lineages. We then used Linear Discriminant Analysis (LDA) based on relative abundances of species-level OTUs to increase class separation within data and enable computational efficacy. This supervised machine learning technique identifies linear discriminants that maximize the separation between the means of groups of data while minimizing the variance within each group of data^32^. The first linear discriminant distinguished individuals based on pregnancy status, the second linear discriminant distinguished smokers from non-smokers, and the third discriminant showed differences between smoker and non-smoker pregnant women. This differential clustering indicates that a pulse and a press each exert a unique influence on the subgingival microbiome, and when they are combined, their effect is not similar to either event.

The subgingival microbiomes of both pregnant women and smokers demonstrated significant commensal depletion. Certain commensals with established associations to periodontal health, notably those belonging to *Neisseria, Veillonella* and *Actinomyces*^33^,^34^, were decreased in both groups when compared to controls, pointing to the deleterious effects of these events on microbial community structure. Furthermore, *Neisseria weaveri* and *Veillonella dispar* were two robust network anchors in non-pregnant nonsmokers; however, they were not influential in pregnant women or in smokers. In these women, the

Smoking appears to promote the growth of anaerobes, while pregnancy appears to promote the growth of gram-negative facultatives at the expense of anaerobes. This finding is in contrast to earlier studies; which demonstrated a preponderance of gram-negative anaerobes in pregnancy gingivitis^13^,^14^. It is possible that the study population in the present study (periodontally healthy pregnant women) contributed to the differences; and suggest that the anaerobe-enrichment seen in pregnancy gingivitis may result more from gingival inflammation than from the effect of female sex hormones.

Another key finding was that smoking enriched the microbiome for pathogens whose growth is promoted by reducing environments, for example, *Filifactor, Fusobacterium, Megasphaera, Aeromicrobium, Dialister* and *Desulfobulbus*, while pregnancy was associated with an enrichment of methylotrophs (*Pseudomonas* and *Methylobacterium*), Streptococci and phenotypically similar species (*Enterococcus, Vagococcus*). Methylotrophs are organisms that obtain energy for growth by oxidizing one-carbon compounds, such as methanol and methane. *Streptococci*, *Enterococcus* and *Vagococcus* share a common ability to metabolize estrogen^35^,^36^,^37^. Methylotrophs have also been previously identified in breast cancer and bacterial vaginosis^38^,^39^. It has been previously demonstrated that the levels of estrogen receptors in the gingiva and estradiol in saliva increase during pregnancy^30^,^40^, suggesting that the findings of the present study are biologically plausible events. *Candida* are also prolific methylotrophs, and their associations with estrogen-rich environments^41^ as well as their contributions to oral health^42^ are well documented. The strong presence of methylotrophs in the present investigations suggests that further studies examining the levels of yeasts in the subgingival microbiome of pregnant women are warranted.

Pregnant women who smoked demonstrated not only enrichment of both methylotrophs and reduction-sensitive species, but also species that ferment mixed acids, for example, those belonging to *Enterobacteriaceae.* It is interesting that these fermentative species utilize methanol as a substrate^43^. Taken together with the finding that nearly half the species that were different between pregnant smokers and controls were unique, the data indicate that effects of pregnancy and smoking on the microbiome are not simply additive, but that the multiplicative effects of a pulse superimposed on a pressed event result in a significantly different microbial profile.

Possibly one of the most interesting findings in this study is derived from an examination of the co-occurrence networks. Bacterial colonization of a niche is primarily driven either by nutritional, spatial or metabolic factors that select for these species. The deterministic theory of species abundance suggests that the environment imposes habitat filters for species that possess traits suitable for that environment^44^. Thus, the presence of strong co-occurrence networks indicates that the environment plays a key role in selecting for the species. We used betweenness centrality of co-occurrence networks to identify species that served as network anchors. Betweenness centrality measures the potential of each graph node (s-OTU) in supervising or influencing the flow of resources and information in a network^45^. Based on this metric, species that connect two or more clusters or consortia rank highly in a network. It has been demonstrated that *Pseudomonas, Prevotella, Methylobacterium, Streptococcus, Megasphaera, Atopobium, Acinetobacter* and *Escherichia* are highly abundant in estrogen-rich environments^46^,^47^,^48^. Their co-occurrence either in a single cluster or as network anchors in only in pregnant women (smokers and nonsmokers) suggests that estrogen might influence their colonization. By extension, these co-occurrences also provide information about little known species that form part of these networks^44^. As a case in point, *Vagococcus* and *Enterobacter* are two species that cluster with the estrogen metabolizers in these individuals, and *Dialister spp.* is a network anchor in pregnant smokers and nonsmokers, however, thus far, little is known about these species. Their strong correlation with species that are known to be affected by estrogen provides a strong indication that these species are also capable of estrogen metabolism. Further studies are warranted to test this hypothesis.

Interestingly, two species that have been famously associated with pregnancy-related periodontal diseases- *Prevotella intermedia* and *Campylobacter rectus*- were not detected either as numerically dominantly members or as significantly different members of the subgingival microbiome of pregnant women. This may be because, as stated previously, our cohorts were periodontally healthy. However, in pregnant women, *Campylobacter spp.* emerged as a co-occurrence influencer, while in pregnant smokers, *Prevotella melaninogenica* and *Prevotella spp.* were strong network anchors. It is possible that their important role as community regulators in pregnant women allows them to emerge as numerically dominant members of the community when inflammation is added to the subgingival milieu.

We are aware that the small sample size as well as the lack of correction for multiple comparisons may have some impact on our findings. However, since this was an exploratory analysis, and the focus was more on community level metrics rather than differences in individual taxa, we did not correct of multiple comparisons. Further studies with larger sample sizes are warranted to validate these findings.

In summary, the subgingival microbiome responds differently to smoking and pregnancy. While smoking causes an increase in anaerobic organisms that thrive in reducing environments, pregnancy appears to promote colonization by species capable of estrogen metabolism. The greatest implications of this study are for women who smoke during their pregnancy, since the shift in the microbiome does not appear to be simply additive effects of pregnancy or smoking, but a multiplicative effect of the combined effects of these two events on the microenvironment. The study thus highlights the importance of pulsed events in altering microbial dynamics. It is important to examine shifts in the microbiome in these women following parturition.

## Additional Information

**How to cite this article**: Paropkari, A. D. *et al.* Smoking, Pregnancy and the Subgingival Microbiome. *Sci. Rep.* **6**, 30388; doi: 10.1038/srep30388 (2016).

## Figures and Tables

**Table 1:** Clinical and demographic characteristics of the study population.

	Non-pregnant nonsmokers(Controls)	Non-pregnantsmokers	Pregnantnonsmokers	Pregnantsmokers
	n = 11	n = 11	n = 11	n = 11
Demographic characteristics				
Age (years ± SD)	21.3 ± 1.3	22.8 ± 2.8	19.7 ± 1.2	22.2 ± 1.9
Ethnicity				
African Americans	9	8	9	9
Caucasians	2	2	1	2
Asians	0	1	1	0
Tobacco exposure (lifetime)				
Smoking history in pack years (± SD)	0	6.3 ± 1.5	0	6.9 ± 0.8
Periodontal characteristics				
Plaque level (% of sites ± SD)	43.1 ± 2.9	38.4 ± 3.4	42 ± 4.6	33 ± 5.2
Bleeding (% of sites ± SD)	24.8 ± 1.7	26.3 ± 4.2	23.1 ± 1.4	23.1 ± 4.2
No. of sites with PD > 4 mm (± SD)	2.6 ± 1.4	2.2 ± 1.1	2.3 ± 0.8	2.4 ± 1.1
Mean CAL (± SD)	0.8 ± 0.3	1.2 ± 0.2	1.1 ± 0.5	0.7 ± 0.1

There were no statistically significant differences (p > 0.05, Tukey HSD) between pregnant and non-pregnant groups for clinical findings as well as age and race distribution.
